# Atypical Song Reveals Spontaneously Developing Coordination between Multi-Modal Signals in Brown-Headed Cowbirds (*Molothrus ater*)

**DOI:** 10.1371/journal.pone.0065525

**Published:** 2013-06-17

**Authors:** Amanda R. Hoepfner, Franz Goller

**Affiliations:** Department of Biology, University of Utah, Salt Lake City, Utah, United States of America; UCLA, United States of America

## Abstract

The courtship and dominance behavior of brown-headed cowbirds (*Molothrus ater*) consists of a multi-modal display, including song as well as postural and wing movements. The temporal sequences of the acoustic and the visual display are coordinated. In adult male cowbirds the largest wing movements of the display are synchronized with silent periods of song, but it is unknown how this coordination emerges during song development. Here we investigate how visual display features are coordinated with song by using atypical song sequence structure of isolation-reared male cowbirds. In birds with atypical song, all components of the visual display were highly similar to those of “normal” song displays, but their timing was slightly different. The number of maximal wing movement cycles of isolation-reared males was linked to the number of sound units in the song, and was therefore reduced during the abbreviated song types of isolates. These data indicate that young cowbirds do not need to be exposed to a model of the visual display during ontogeny and that there is synchronization with the temporal structure of song. A physiological link between respiratory and syringeal control of silent periods between sound units and wing movement cycles may be driving this synchronization.

## Introduction

Many animals make simultaneous use of different sensory channels for communication. For example, birds frequently combine acoustic and visual signals in their elaborate territorial and courtship displays. This multi-modal signaling is a common behavioral feature of members of the blackbird family (Icteridae) whose visual display often contains dynamic movements (e.g. [1]–[4]). However, elaborate postures and movements are likely to affect the movements associated with song production such as respiration and upper vocal tract filtering (e.g. [1], [2], [5], [6]). These simultaneous performances may require coordination between these potentially interfering display components. It is largely unknown how this coordination develops.

**Figure 1:**
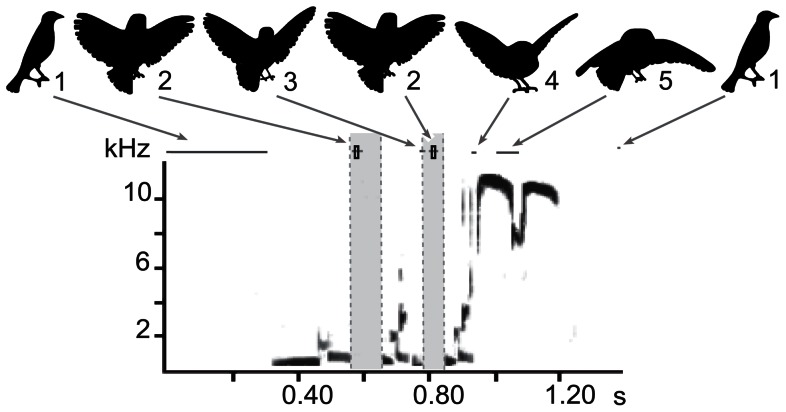
Coordination of visual display and song in a socially raised cowbird. Song and display are coordinated in socially raised cowbirds. A spectrographic representation of a song type with 3 sound units, which are separated by silent periods (grey between the dashed vertical lines). Above the spectrogram, frames of cartoon versions of the bird's outline taken from simultaneously recorded high-speed video of the displaying bird are shown. Ranges of various defining positions of the visual display are indicated above the spectrogram (grey, horizontal lines) and the mean (black, vertical boxes).

In the brood-parasitic brown-headed cowbird (*Molothrus ater*), the combined displays are thought to function in courtship as well as in inter-male communication (e.g. [3], [7]). The visual display consists of postural changes (puffing), which precede and accompany song; multiple wing movements performed along with tail spreading during the song and a bowing movement that occurs at the end of the song. In adult males, the most elaborate wing movements are synchronized with the silent periods during the song ([2]; Figure 1). The unfolding of the wings begins prior to, or right at the onset of the song (silhouette labeled 1). Typically the wings are unfolded into a low position (from here on “low”; silhouette label 2) during the first silent period before being lifted to a high wing position (from here on “high”; silhouette label 3) during the following expiratory pulse. The number of low to high wing movements is correlated with the number of expiratory pulses in the song. Typically, the last high to low wing movement occurs simultaneously with a bow during which the wings are folded (silhouette labels 4 and 5). The wing display can be highly variable in regard to how far the wings are extended and how much they are moved from an elevated backward position to a lowered forward position. The intensity of this wing display depends upon the audience. Female directed solicitation is accompanied by smaller wing spread displays than male directed intimidation displays [3].

Male cowbirds sing a number of distinct song types [8], each of which is produced with 3–4 expiratory pulses [2], [9], [10] that give rise to the temporal sequence of song. The first pulses are used to generate introductory note clusters that are characterized by alternating contributions from the two syringeal sound sources. The last expiratory pulse gives rise to a high-frequency “whistle” that is generated on the right side of the syrinx [9]. Each expiratory pulse of song (from here on “sound unit”) is followed by a short, deep inspiration (minibreath), which coincides with silent periods of the song. Interestingly, the silent periods during which wing movements occur are prolonged by a period of silence that arises as the syrinx is held closed and expiratory pressure is built [9]. This generation of silent periods through closure of the syrinx, while expiratory pressure is at a phonatory level, is highly unusual in songbirds [6], [11]. Its presence in cowbirds suggests that this mechanism is used to prolong the silent period between note clusters to avoid interference of biomechanical effects of wing movements on stereotyped song production [2].

This clear synchronization between aspects of the visual display and song poses the intriguing question of how this coordination between different movements develops. Cowbirds are oscines and therefore vocal learning plays a role in the development of song, although in this species the basic song structure may arise without exposure to conspecific song (e.g. [12]–[14]). In contrast, the ontogeny of the visual display has not been studied in detail, and it remains unclear how the synchronization between the visual display and the song is established during development. It is not known if male cowbirds require a model for developing a typical visual display, although reports on songs of isolate cowbirds do not indicate the absence of a visual display (e.g. [12]).

As a first step toward understanding the synchronization of visual and vocal displays, we make use of abnormal song sequences of male cowbirds reared in isolation and assess how the visual display is coordinated with these atypical songs. Could the respiratory movements of song provide a template for the wing movement pattern of the visual display? The results indicate that the wing movements of the visual display were adjusted to the altered song sequence, suggesting that the acoustic motor program guides the timing of the main movements of the visual display.

## Methods

We raised five nestling male cowbirds by hand in isolation from visual and acoustic contact with adult conspecifics (from here on “isolate male”). The nestlings were collected at Konza Prairie Biological Station (Kansas) in July 2007. Their display behavior was compared to that of five males that were caught as adults and, judged by adult plumage, were at least 2 years old at the time of collection (from here on “socially raised male”). Four of the adults were caught during the 2007 breeding season at Farmington Bay Waterfowl Management Area (Utah), and one bird was caught during the winter in Fort Hood (Texas). The nestlings were collected from the nests of parasitized hosts and the adults were collected by mist nets. Collection of birds was approved by United States Fish and Wildlife Services and by each respective state. All of the animal research was approved by the Institutional Animal Care and Use Committee at the University of Utah.

The isolate males were less than 10 days old when they were collected. After fledging, they were initially housed in nestling groups for 24 days until they no longer needed to be fed by hand. They were then housed singly in a cage (34×38×40 cm) that had a Plexiglas front and acoustic foam on all of the other sides to prevent echoes. All of the birds were faced so that they were unable to see one another and were kept at a photoperiod that matched that of Salt Lake City, UT. The isolate males were recorded in their first year in July 2008, when they were about twelve months old. Two of these birds were recorded again in their third year, one after being housed singly in a cage during the second and third year in an aviary that permitted acoustic and visual contact with wild-caught, adult males and females, the other after continued isolation from conspecifics.

Each male was placed in a recording box (63.5×51×51 cm) accompanied by a wild-caught, adult female to stimulate song and display behavior. We also used adult males to stimulate singing behavior for birds older than 2 years. The front wall of the recording box consisted of clear Plexiglas to allow filming and was lined with acoustic foam to decrease echoes. We used a (HSC 250×2 by JC Labs Inc.) high-speed video system to film the birds at 250 frames per second and digitized the video using Pinnacle Studios (v. 12). For the analysis we used a frame rate of 125 frames per second. Along with the video, sound was recorded with an omnidirectional microphone (Audio-Technica AT3032) that was inserted into a hole in the roof of the recording box. We recorded the audio recordings in real time using Avisoft Recorder (v. 3.4). We used frame-by-frame analysis to align components of the visual display with the song for each individual.

Cowbird song consists of sound units that are separated by silent periods. We classify these sound units into 3 groups; introductory long- duration low frequency sounds (unit 1), introductory note clusters consisting of rapidly alternating notes of different frequency (unit 2) and a combination of alternating low-frequency notes with a high-frequency whistle (unit 3). This last sound unit is typical of the subspecies *Molothrus ater artemisiae* (seen in the social and isolate males) and differs from that of other subspecies (e.g. [15]) by containing low-frequency elements in the last sound unit. Typical songs consist of the sequences 1+2+3 or 1+2+2+3.

The puffing and bowing components of the visual display in isolates were compared qualitatively to those of socially raised birds. These components vary substantially in normal display behavior within and between different song types and are difficult to quantify precisely, because they consist of slow, gradual changes in movement. Furthermore, viewing angle affects apparent size changes during the “puffing” component of the visual display, making measurements unreliable. Although we did not quantify all display features, qualitative assessment did not reveal a difference between isolate and socially raised birds. Wing movements, however, can be reliably quantified, and they contain rapid movements with demonstrated effects on song production [2]. Songs with the most elaborate wing display were chosen to allow precise determination of timing. If available, we selected at least 5 renditions per song type for each individual. Because the lowest wing position could be most reliably identified in the frame by frame analysis, we selected this aspect of the wing display for synchronization analysis with song. The lowest position was defined as the point where the wings stop extending forward (or down), i.e. the subsequent frame shows a reverse movement. During the last sound unit as the wings started to close, some individuals went through an additional low wing position. However, whether or not a distinct low position could be detected varied greatly within and between individuals. These variable events are not included in the quantitative analysis.

The duration from the lowest wing position to the beginning of the following sound unit was measured. The duration of silent periods varies between different song types and individuals. To normalize for this variation, we calculated the timing of the lowest wing position as a fraction of the duration into the silent period during which it occurred and expressed it as a percentage. This allowed us to compare the timing of the lowest wing movement across song types and individuals. The measurements of lowest wing position were also labeled according to the silent period of the song with which they were associated according to the following convention: Low3 corresponds to the silent period preceding the last sound unit (i.e., the last expiratory pulse or the whistle). Low1 corresponds to the wing position associated with the first silent period, if multiple silent periods are present. Low2 is the wing position associated with the second silent period, if 3 are present (i.e., song is comprised of 4 sound units). To compare the differences in wing placement of the isolates and socially raised males, we computed the mean percentages for each measurement (Low1, Low3) and tested for differences using a nested ANOVA. The variance was partitioned into song type, individual and group (socially raised versus isolate) (using Minitab 16.1.1 for Windows). Low2 did not have a large enough sample size to run an ANOVA, thus these measurements were only used when examining the low position as a whole.

## Results

**Figure 2:**
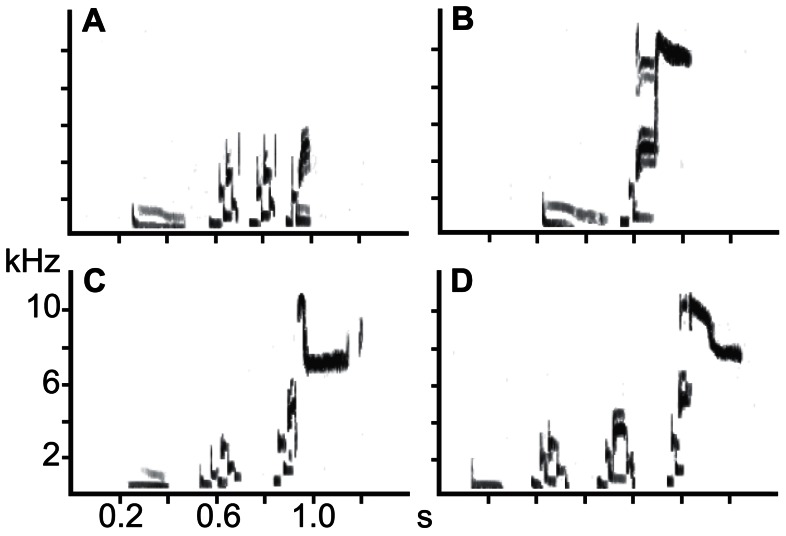
Songs of isolate cowbird before and after social exposure. Spectrograms of songs from CB14 who was recorded in his first singing season (a, b) and re-recorded in 2010 after exposure to adult male and female cowbirds (c, d). Songs in the first year consist of atypical sound sequences (a. units 1+2+2+2; b. units 1+3), whereas after social experience the same bird sang “normal” sequences (c. 1+2+3; d. 1+2+2+3).

**Figure 3:**
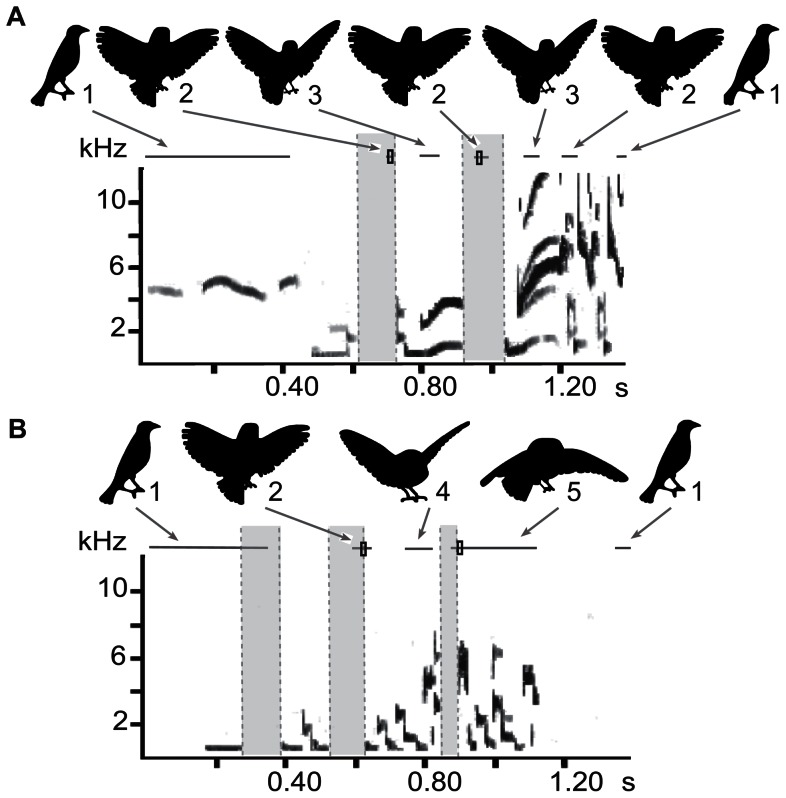
Coordination of visual display and song in isolate cowbirds. Isolate males sang “abnormal” songs with “normal” coordinated visual displays. Two examples with spectrograms and corresponding silhouettes of display features are shown (a. CB18 first year, 3 sound units with aberrant acoustic structure; b. CB10, third year, 1+2+2+2). The various components of the visual display are all present and appropriately positioned during song. The number of full wing movement cycles is adjusted to the number of sound units.

The song types of isolate birds deviated from the typical song sequence of this subspecies (Figures 1–3). Either they contained fewer, acoustically abnormal sound units, and/or the song consisted of an incorrect sequence of sound units. For example, the last sound unit of a song type containing four sound units consisted of alternating low-frequency sound and did not include a whistle (1+2+2+2; Figure 2a). Other song types consisted of only 2 sound units (1+3; Figure 2b). It is important to note that this atypical song structure was not a simple, age-related imperfection in song, because isolate males retained these song types in their second year (one male was also recorded in the third year). The only exception was the song of CB14, following housing in years 2 and 3 with socially raised cowbirds. After this acoustic experience, his song types contained a subspecies-typical sound unit structure and the typical number of sound units (Figure 2c, d). In general, the song structure of isolate birds showed clear differences that allowed us to test whether display features develop with or separate from song.

**Figure 4:**
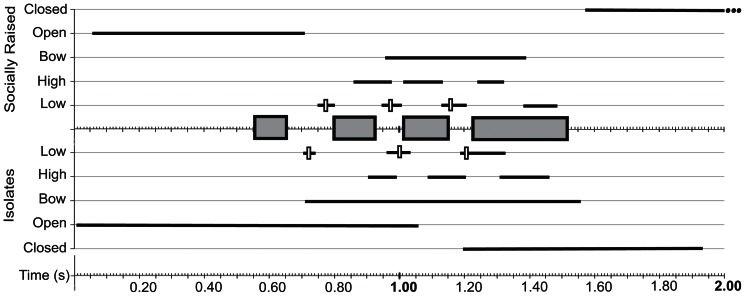
Summary of coordination between song and visual display for songs with 4 sound units. Temporal synchronization between components of the visual display and songs composed of 4 sound units is similar in socially raised (top) and isolate (bottom) cowbirds. To represent the temporal structure of song, the mean duration of sound units (grey box) and intervening silent periods is depicted for all song types with 4 sound units from all birds. No distinction was made in regard to which of the 3 sound units was present at what position, and, as described in the text, these units varied between socially raised and isolate birds. The time during which various components of the visual display occur relative to the temporal segments of song is shown by horizontal lines (full range of all observed occurrences). For “low” position of wing, the component that could be determined with the highest temporal precision, the mean is indicated by the vertical open box. Songs were accompanied by maximally 3 full wing movement cycles. An occasional fourth low position was quantified before closure of the wings at the end of the wing display but was not followed by an upward movement.

**Figure 5:**
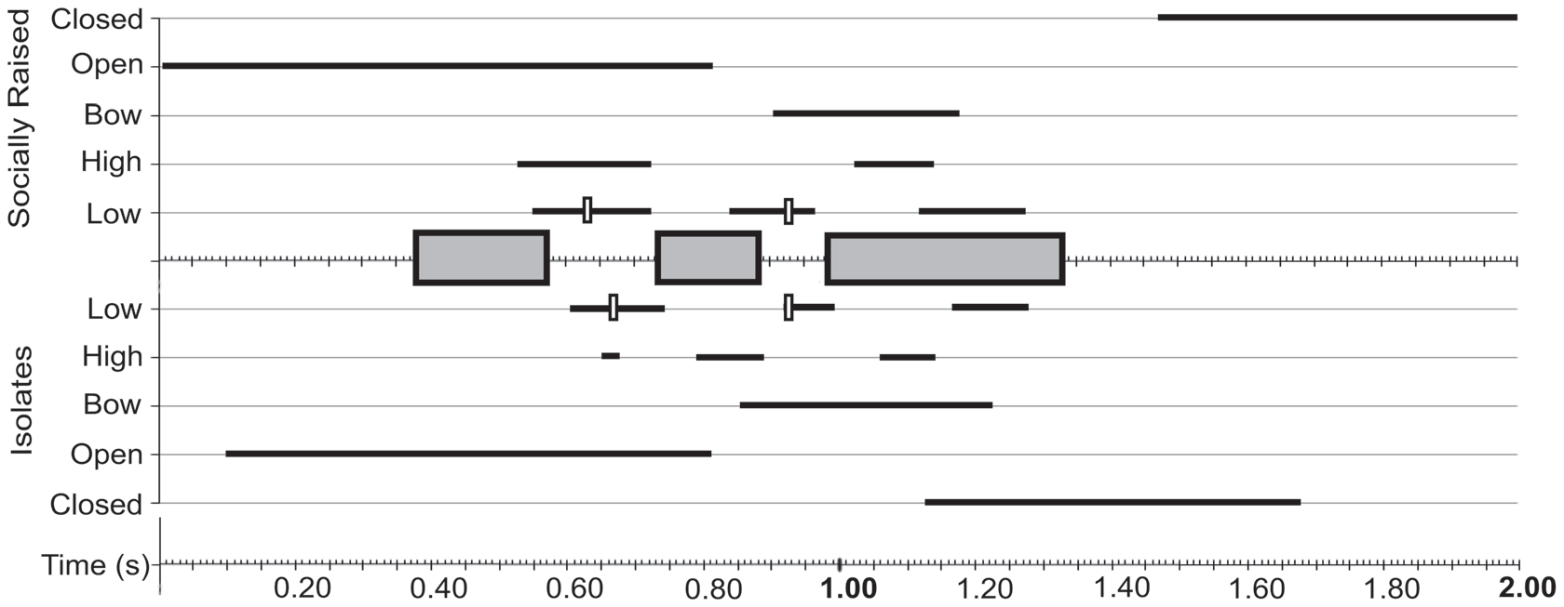
Summary of coordination between song and visual display for songs with 3 sound units. Songs composed of 3 sound units were also accompanied by similar displays with similar coordination. The units of sound were accompanied by maximally 2 full wing cycles. Data are displayed as described for Figure 4.

Isolate birds showed all elements of the visual display: puffing, wing opening, up and down movements of the wings, wing closing, tail spreading and bowing (Figure 3). Several differences were present in the timing of visual display aspects between the two groups. For example, isolates tend to open the wings later in relation to song (see below). In addition they begin to close their wings earlier than socially raised cowbirds, who typically closed their wings after the conclusion of the song. Also, the range of when isolates start to bow is broader, and bows occur slightly earlier. The extent of wing opening and the degree of wing movements is also highly variable, even within different renditions of the same song type within an individual. The range of variation in intensity of opening was similar for isolate and socially raised birds (Figures 4, 5). Because of this large variation it was difficult to quantify aspects of the visual display; therefore we focused on the most easily quantified aspect, the extreme position of the wings during the wing movements.

**Figure 6:**
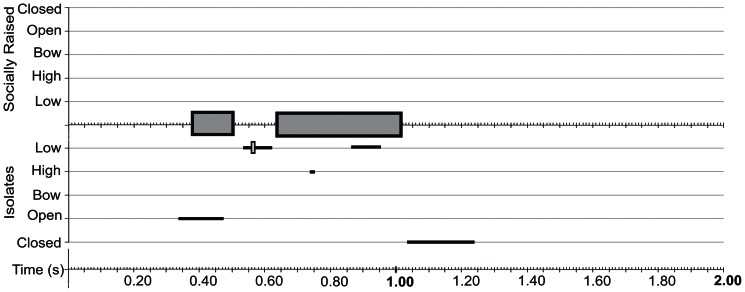
Summary of coordination between song and visual display for songs with 2 sound units. Isolate birds sang songs composed of only two sound units, but socially raised males did not sing such song types. Despite the abnormal song sequence, components of the visual display were coordinated with the shortened acoustic signal, and only one full wing movement cycle was performed. Data are displayed as described for Figure 4.

The atypical songs of isolates provide the opportunity to test whether and how wing movements are linked to song structure. Despite the deviation in the temporal sequence of song, the number of wing movement cycles of isolate birds was matched to the variable song duration in a way similar to that of socially raised birds. In other words, if songs contained fewer sound units, the duration of the visual display was also shorter (Figures 4–6). This is most easily seen in the number of up-down wing movement cycles, which were adjusted to the number of silent periods between sound units. Songs consisting of four sound units in isolates (1+2+2+2) were accompanied by a visual display with three full wing cycles (Figure 4), whereas displays of songs with 3 units (1+2+2) had maximally 2 full cycles (Figure 5). Abnormal song sequences, which consisted of only 2 units (1+3) and do not occur in socially raised birds, were accompanied by a visual display with only one full wing cycle (Figure 6).

**Figure 7:**
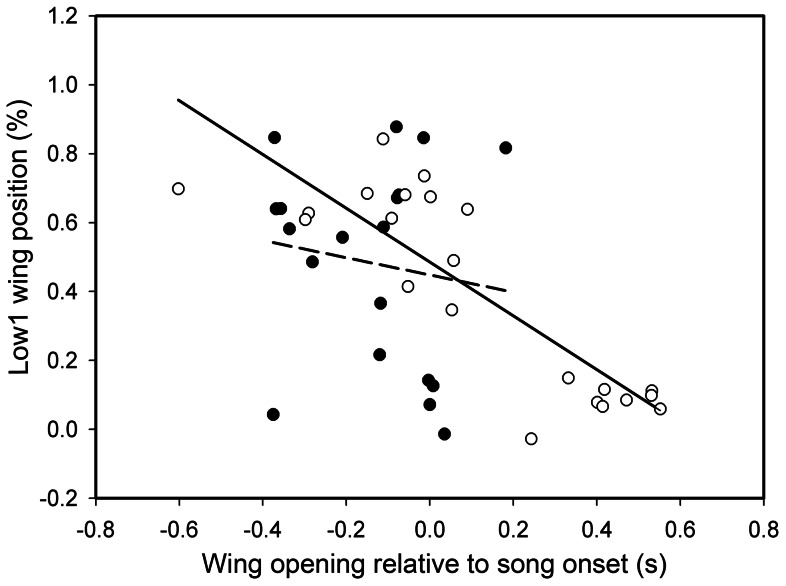
Low1 placement is related to the timing of wing opening in isolates but not socially raised birds. The relation of the beginning of the wings opening was measured relative to the onset of song (negative numbers indicate prior to song). Wing opening and Low1 position were not related in socially raised cowbirds (open circles) (regression, dashed line, R^2^ = 0.02, regression: F = 0.33; p = 0.57). In isolate birds Low1 occurred later with later opening of the wings (black circles, regression, solid line, R^2^ = 0.72, regression: F = 51.9; p = 0.0001).

The lowest wing position occurred during or at the end of the silent periods (Table 1; Figure 1, 3–6), thus assuring that the largest wing movements typically take place during the silent period. The mean placement of all measurements for the lowest wing position was at 42.4±6.1% (n = 56) into the silent period in isolate birds and at 56.2±3.7% (n = 70) in the socially raised birds (the difference was significant, nested ANOVA: F = 5.53; p = 0.02). The placement was also significantly different between individuals (nested ANOVA: F = 2.02; p = 0.048), however, not between song types (nested ANOVA: F = 1.53; p = 0.13) In separate nested ANOVAs for each low position, Low1 placement showed a significant difference for group, individual, and song type (nested ANOVA for all three factors: p = 0.0001); whereas the Low3 placement was only significant for individual (nested ANOVA: group F = 2.66; p = 0.11, individual F = 2.18; p = 0.04, song type F = 1.26; p = 0.27). This difference is partly caused by the timing of the onset of the wing opening. The timing of the Low1 wing position was related to the timing of the initial unfolding of the wings (Figure 7). In isolates, the timing of Low1 was significantly linked to when wing opening occurred (R^2^ = 0.72, F = 51.9; p = 0.0001). This relationship was not seen in socially raised cowbirds (R^2^ = 0.02, F = 0.33; p = 0.57). Overall, the placement of the low wing movement was typically in the silent period. Only in two isolate cowbirds, CB14 and CB10, was Low3 placed at the beginning of the following sound unit (Figure 3b, Table 1). Interestingly, the tendency in isolated males to place the lowest wing position later in the silent period (indicated by the lower percentage, Table 1) was present in CB14 before social exposure to conspecifics, but changed to the typical placement (42.45% vs 67.68%; Table 1) after his song was modified to the correct unit sequences in year 3. Neither of these two changes occurred in CB10 who was left in social isolation.

**Table 1:** Timing of lowest wing position within silent periods for individual song types.

Group	Individual	Song	Sample Size	Silent Period1	Low1	Percent1	Silent Period2	Low2	Percent2	Silent Period3	Low3	Percent3
Socially Raised	CB3	A	3	159±3	99±2	62.2±1.8	-	-	-	103±1	48±5	46.9±5.2
	CB40	B	2	162±10	-	-	159±18	73±47	49.5±34.8	73±3	54±35	76.1±51.1
	CB2	C	5	93±6	73±7	77.8±4.3	-	-	-	88±1	35±6	39.4±6. 5
		D	5	144±2	24±9	16.7±6.2	112±2	44±14	39.7±12.2	94±1	76±5	80.3±5.3
	CB4	E	5	128±12	65±16	41.7±11.3	-	-	-	106±3	44±2	41.7±3.0
		F	4	126±4	132±11	104.6±7.1	-	-	-	105±1	50±7	47.5±7.2
	CB7	G	4	150±3	-	-	-	-	-	114±2	79±28	69.1±24.5
		H	6	153±3	-	-	115±1	40±9	34.9±7.9	117±1	105±7	89.9±5.9
		T	2	110±2	-	-	-	-	-	113±2	42±12	37.0±9.9
Isolates	CB18	I	5	283±3	16±9	5.7±2.7	-	-	-	111±1	64±2	57.6±2.2
		J	5	290±2	29±4	10.1±1.5	-	-	-	116±1	75±10	64.6±8.8
	CB14a	K	3	102±2	50±9	49.1±8.4	48±4	-	-	43±1	24±11	56.6±26.7
		L	4	-	-	-	-	-	-	163±2	114±24	68.9±8.2
		U	2	101±4	-	-	-	-	-	63±4	−3±1	−4.9±1.9
	CB14b	M	3	121±6	78±1	64.5±2.6	-	-	-	123±5	89±25	72.9±19.9
		N	3	143±5	102±13	71.2±6.9	-	-	-	146±5	91±5	62.0±3.9
	CB28	O	4	154±4	97±12	62.6±7.2	-	-	-	137±5	46±5	33.7±3.9
		P	3	-	-	-	-	-	-	105±8	38±6	36.6±6.5
	CB23	Q	3	85±2	-	-	-	-		90±2	63±11	70.4±11.8
		R	3	222±6	-	-	35±5	10±15	15.8±45.9	37±1	-	-
	CB10	S	5	110±2	-	-	104±1	5±13	4.9±12.2	52±2	−1±25	−3.9±50.4

The occurrence of the wing low position within the silent period is represented as a percentage of the duration of the silent period from the following sound unit. Negative percentage values indicate that the lowest wing position occurred during the following sound unit. CB14a and b list the measurements for song types of the same bird recorded in 2008 (a) and in 2010 (b).

## Discussion

The visual displays of isolate birds contained all components of the typical display of brown-headed cowbirds. The degree to which certain components, such as the wing spread and movements are expressed can vary substantially from song to song and with the audience [3]. No major qualitative differences in these components were observed between isolate and socially raised birds. This indicates that the sequence of visual display components develops without previous exposure to displaying males. Despite these similarities in the main components of the display, however, subtle differences may exist between displays from isolate and socially raised birds, and even small differences may be relevant for communication. The communicative function of the different components of the display is largely unknown. More elaborate displays are performed towards other males, whereas those directed at females contain less dramatic wing spreads and movements [3], suggesting that visual signals may be more important for intra-sexual communication. Nevertheless, the respective potency of individual display components remains to be explored.

The atypical sequence of sound units in the songs of isolate males provided a good test for how the visual display is integrated with song. Because the number of maximal wing movement cycles is adjusted to the number of sound units in the song of different song types of socially raised birds, one might expect that the displays develop through a coordination mechanism. Atypical songs were accompanied by the “correct” number of wing movements, even if the number of sound units was less than the lowest number found in songs of socially raised birds. This result indicates that this coordination is achieved without exposure to either song or visual displays. Because experience with conspecific displays is not necessary for developing this coordination, song structure appears to provide a “guiding” template for the wing movements of the visual display. It is unclear whether a stereotyped coordination for each song type develops during the sensori-motor practice period, such that later the coordination of motor sequences is as fixed as the motor sequences of song, or whether the coordination is employed at the motor planning stage at each performance of the song.

Wing movements are likely to affect respiratory movements during song. Placing the most elaborate movement (from high to low wing position) within the silent period may be energetically favorable [2]. The extension of the silent period, as discussed above, may therefore accommodate the wing movements of the display [2] and, thus pose constraints on possible synchronization of visual and vocal display features. Consistent with this idea is the fact that elaborate wing movements occur only during the prolonged silent periods and not during “normal” silent periods consisting only of a minibreath. This is illustrated by the different song structure in *M. ater ater* and *M. ater ateremisiae*. The last sound unit in the songs of the former subspecies contains only a high-frequency whistle and the silent period before this unit consists only of a minibreath [9]. The last unit in songs in *M. ater artemisiae* are composed of a note cluster portion and the high-frequency whistle, and the preceding silent period consists of a minibreath and expiratory silent period ([10], shows a song of *M. ater ater* in Figure 2 and a song of *M. ater artemisiae* in Figure 8; [2]). This difference suggests that the occurrence of an expiratory silent period is linked to a note cluster sound sequence. Unlike *M. ater artemisae*, males of *M. ater ater* do not perform a wing movement cycle during the last silent period that does not contain an expiratory silent period and is not followed by note cluster sound element (Goller, unpubl. observation).

The findings from isolate birds and differences between subspecies suggest that wing movement cycles are linked to prolonged silent periods in connection with note cluster elements of song. The motor sequence for the active closing of the syringeal valves during expiratory pulses may be a defining event for the coordination between the motor programs for the wing movements and the song. However, the precise timing of the wing movement could depend on social cues, because isolate birds tended to place the wing movement cycle somewhat later in the silent period than socially raised birds. Further support for the link between the movements of vocal and visual displays during song can be derived from the observation that the wing movements remain correlated with the song sequence, if song changes from year to year.

In conclusion, we show that the movements of the visual display are coordinated with song irrespective of the song sequence. This coordination emerges without exposure to displaying adult males. We suggest that motor actions effecting an atypically prolonged silent period between sound units may constitute the defining events that could provide timing information for coordination of the motor programs of the visual display and song. Cowbird males that display during a song elicit a stronger response from females than song alone [4]. Increased complexity of signals and their integration provide additional information about the sender and his potential for precise motor coordination. This strong correlation between timing of wing movements and silent periods suggests strong evolutionary pressures for the integration of song and display.
